# Probing Surface Changes in Fe–Ni Oxide Nanocatalysts with a ToF-SIMS-Coupled Electrochemistry Setup and Principal Component Analysis

**DOI:** 10.1021/acs.analchem.5c03894

**Published:** 2025-12-16

**Authors:** Heydar Habibimarkani, Jörg Radnik, Vasile-Dan Hodoroaba, Elisabeth John

**Affiliations:** 42220 Federal Institute for Materials Research and Testing (BAM), Unter den Eichen 87, Berlin 12205, Germany

## Abstract

Understanding catalyst surface dynamics under operating conditions is essential for improving electrocatalytic performance. Here, we present a novel approach combining electrochemical treatment with contamination-free transfer to Time-of-Flight Secondary Ion Mass Spectrometry (ToF-SIMS), followed by principal component analysis (PCA), to probe surface and interfacial changes in Ni–Fe oxide nanoparticles stabilized by polyvinylpyrrolidone (PVP) during the oxygen evolution reaction (OER). The surface analysis at three distinct treatment stages revealed distinct chemical fingerprints across pristine nanoparticles, after exposure to 1 M KOH electrolyte, and after cyclic voltammetry treatment. The results highlight a progressive transition from ligand-rich to ligand-depleted interfaces, with PVP-related fragments dominant in the early stages and metal- and electrolyte-derived species emerging after activation. Complementary ToF-SIMS analysis of electrolyte deposited on Si wafers after each treatment step confirms the concurrent leaching of PVP and Fe–Ni-based fragments during OER. These findings underscore the dynamic nature of catalyst–electrolyte interfaces and demonstrate a robust strategy for monitoring surface-sensitive chemical changes associated with the nanoparticles, especially during the initial cycles of the OER.

## Graphical abstract

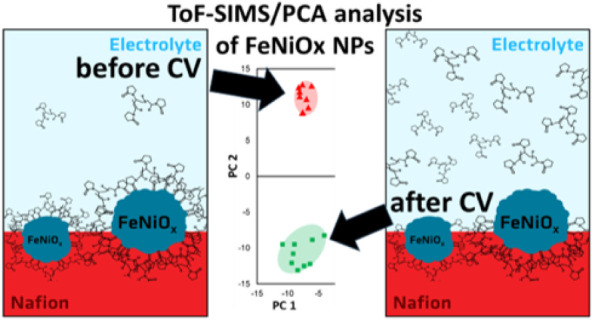

## Introduction

The global shift toward sustainable energy has intensified research efforts into electrochemical water splitting, particularly the oxygen evolution reaction (OER), which remains a kinetic bottleneck due to its sluggish four-electron mechanism. While noble metal oxides such as IrO_2_ and RuO_2_ exhibit high OER performance, their scarcity and high cost hinder large-scale implementation. As alternatives, earth-abundant transition metal-based nanomaterials, especially Ni–Fe oxides, have emerged as promising candidates owing to their synergistic redox chemistry and high catalytic activity in alkaline media.^1–4^

However, achieving optimal OER performance is not merely a matter of bulk composition. The electrochemically active surface, shaped by ligand coverage, leaching, defect dynamics, and interface reconstruction, plays a decisive role in dictating catalytic behavior.^5–7^ Akbashev emphasized that oxide surfaces are far from static; they evolve continuously under reaction conditions through cation leaching, surface amorphization, and oxygen vacancy formation, creating a dynamic interface that cannot be captured by conventional bulk techniques.^8^

A critical aspect of nanoparticle-based catalysts is the role of surface ligands—such as polyvinylpyrrolidone (PVP)—commonly employed for nanoparticle stabilization during synthesis.^9–12^ These ligands, while necessary during fabrication, often hinder electrochemical performance by blocking active sites. Electrochemical cycling has been shown to enable their effective removal via the formation of strong metal–oxygen or metal–hydrogen bonds, displacing the original ligand–metal coordination without altering particle morphology. This process not only exposes the active surface but also leads to a significant improvement in electrochemical surface area and catalytic activity, underscoring the importance of electrochemical pretreatment in catalyst activation.^13–15^

These surface ligands are not simply inactive residues. Instead, they can actively influence catalytic behavior by affecting charge distribution, reactant binding, and surface hydrophilicity.^16–19^ These findings emphasize the need for carefully controlled ligand removal strategies, such as electrochemical^20^ or thermal treatments,^21^ to ensure optimal exposure of active sites. To fully capture and understand these surface-level changes, surface-sensitive techniques are necessary for revealing the chemical and structural transformations that occur during and after ligand displacement.

Kong et al. highlighted the mechanistic roles of ligands in tuning the electronic structure and steric environment at catalytic surfaces, thereby breaking scaling relationships and enhancing activity and selectivity.^22^ Their work supports the perspective that when not fully removed, ligands such as PVP can still interact with active sites and modulate the electrochemical interface by influencing charge redistribution and double-layer properties.

Time-of-Flight Secondary Ion Mass Spectrometry (ToF-SIMS) provides the high surface sensitivity and chemical specificity needed to analyze changes at electrocatalyst surfaces, particularly those arising from ligand interactions and electrochemical treatment.^23–27^ Zheng et al. employed 3D ToF-SIMS combined with ^18^O/^16^O isotope exchange to spatially resolve oxygen incorporation at buried oxide interfaces, revealing >100-fold enhancement in surface exchange kinetics at heterojunctions compared to bulk regions.^28^

In our study, we apply an advanced electrochemical treatment coupled directly with ToF-SIMS, followed by principal component analysis (PCA) to investigate the chemical dynamics of Ni–Fe oxide nanocatalysts during OER. This integrated approach allowed us to monitor surface changes across distinct catalyst states: pristine, electrolyte-exposed, and post-OER activation. Characteristic fragments from the ligands, polymer matrix and nanocatalyst could be identified. PCA is widely employed in the processing of large data sets to reduce complexity and enhance the efficiency of ToF-SIMS data interpretation.^29–31^ Inspired by the methodology of Heller-Krippendorf et al.,^32^ we extract group-specific characteristic spectra from PCA models, capturing the most functionally relevant surface ions while suppressing background signals.

Recent work by Chen et al. further supports this analytical framework. Their review on component leaching emphasizes that metal dissolution and redeposition—particularly of Fe and Ni—are thermodynamically driven processes that reshape catalytic surfaces during operation. They recommend ICP-OES and spectroscopic tools like XPS, Raman, and XAS to monitor such transformations.^33^ In our approach, we demonstrate that ToF-SIMS, due to its high surface sensitivity, is particularly well suited to address these questions concerning interfacial dynamics under electrochemical conditions.

To the best of our knowledge, this is the first study to apply an electrochemical setup directly coupled to ToF-SIMS, combined with PCA-based multivariate analysis, to monitor ligand (PVP) removal, ionomer (Nafion) signal variation, and nanoparticle surface chemical evolution in Ni–Fe oxide catalysts under OER conditions. This integrated approach enables chemical fingerprinting of catalyst surfaces and associated electrolyte modifications across different electrochemical states (pristine, KOH-exposed, and postactivation), while preserving surface integrity through contamination-free transfer. By extracting group-specific ion profiles via PCA, we demonstrate a strategy for deconvoluting complex surface dynamics in electrocatalysis.

## Materials and Methods

### Electrode Preparation and Catalyst Deposition

The Fe–Ni oxide nanocatalyst, synthesized and characterized in our earlier study,^1^ with an Fe:Ni ratio of 2:3, was selected for further characterization by ToF-SIMS analysis using the electrochemical cell (see details later), due to its superior catalytic performance compared to other ratios.

For sample preparation, copper tape was first cut to match the dimensions of a 6 mm diameter glassy carbon (GC) electrode and affixed to the sample holder (Figure S1) to ensure electrical conductivity. The GC electrode was placed on top of the copper tape, and its edges were secured using carbon tape to provide mechanical stability and to suppress edge currents during cyclic voltammetry (CV) measurements. The assembled holder was then mounted onto the electrochemical cell stage (Figure S2), which holds the sample holder and enables its transfer into the main chamber of the ToF-SIMS system for surface analysis.

The catalyst ink was prepared following previously established protocols.^1^ A dispersion was prepared by mixing 2.2 mg of catalyst with 49 μL ethanol, 49 μL Milli-Q water, and 2 μL Nafion, followed by 30 min ultrasonication at 25 °C to ensure homogeneity. Two 10 μL aliquots were drop-cast onto a 6 mm diameter GC electrode, dried at room temperature for 15 min, and stored under vacuum in a desiccator. The deposited catalyst film covered a geometric area of 0.28 cm^2^.

## Experimental Procedure

Surface analysis was performed at three different stages to capture real-time changes in surface chemistry due to electrolyte exposure and electrocatalytic activation.

- **Pristine:** The prepared electrode was directly transferred to the ToF-SIMS system for initial surface characterization.
- **After Exposure to Electrolyte:** The electrode was immersed in 1 M KOH electrolyte for 1 h under an argon (Ar) atmosphere, followed by rinsing with Milli-Q water and drying under Ar flow before ToF-SIMS analysis. The sample was exposed to electrolyte only inside the electrochemical cell; all subsequent rinsing, drying, and transfer steps were performed under inert argon with vacuum-sealed transfer, without further air exposure.
- **After Cyclic Voltammetry Treatment :** After electrochemical testing, the electrode was again rinsed with Milli-Q water and dried under Ar prior to final surface analysis.

In summary, ambient air exposure was limited to the initial loading of the pristine electrode, whereas all subsequent transfers were carried out exclusively under vacuum or inert argon conditions.

### Electrochemical Cell Integration and Sample Transfer

An electrochemistry cell, purchased from SPECS (SPECS Surface Nano Analysis GmbH, Berlin, Germany), was integrated with the ToF-SIMS system to enable controlled electrochemical treatment under argon gas conditions and contamination-free transfer of the sample into the analysis chamber.

Figure 1 presents an external view of the electrochemical cell, showing its interface with the ToF-SIMS system. The core of the setup is a central glass vessel (Figure 1, #3). The vessel is coupled to a buffer chamber (Figure 1, #10) via a flange, allowing vacuum-sealed sample transfer between the electrochemical cell and the ToF-SIMS analysis chamber.

**Figure 1:**
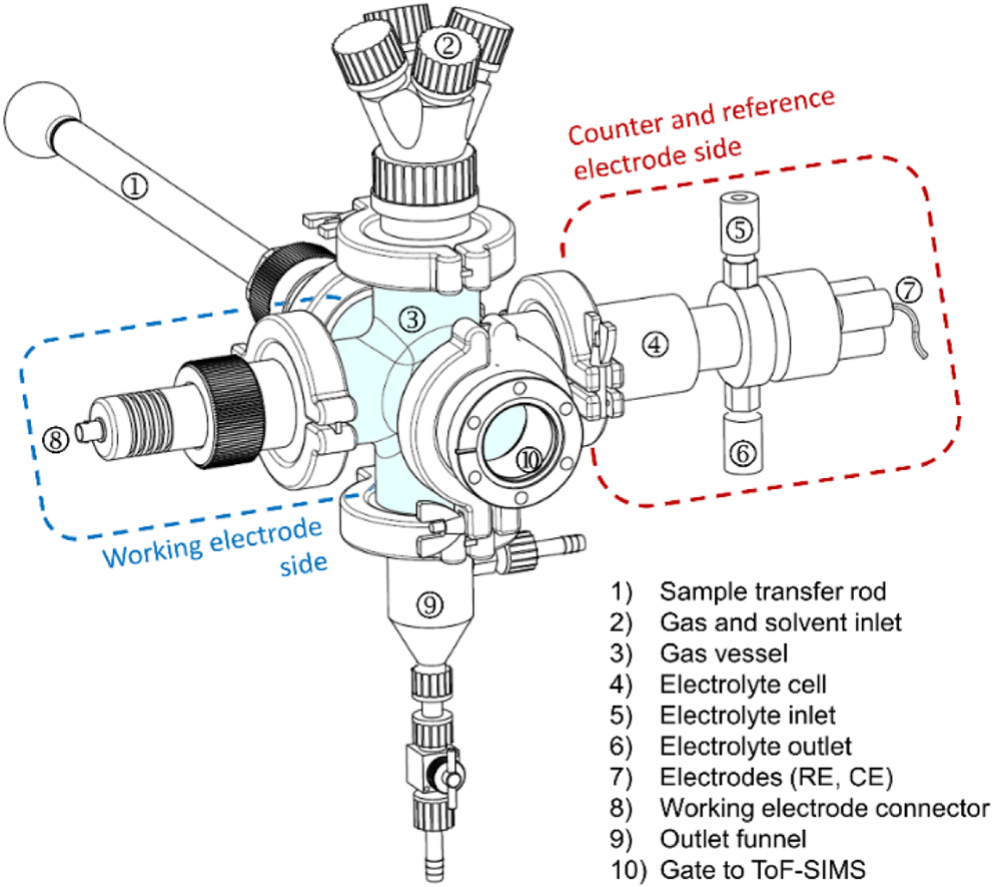
Schematical drawing of the electrochemical setup connected to ToF-SIMS via a gate (#10).

A PTFE (polytetrafluoroethylene) lip seal ensures that only the electrode-facing side of the sample is exposed to the electrolyte, effectively minimizing contamination. The working electrode (WE) is mounted on a spring-loaded sample stage with a connector (Figure 1, #8), providing a stable and reliable electrical contact. On the opposite side, the reference electrode (RE) and counter electrode (CE) are inserted through tight fittings (Figure 1, #7).

A dedicated top flange (Figure 1, #2) enables argon purging, solvent rinsing, and drying. Argon is introduced to purge the system and eliminate oxygen, preventing surface oxidation, while Milli-Q water is used for washing and hydrating the catalyst surface. The electrolyte cell (Figure 1, #4) houses the electrolyte solution required for electrochemical reactions. It is filled via an electrolyte inlet (Figure 1, #5) and drained through an electrolyte outlet (Figure 1, #6). Residual liquid is removed via the bottom outlet funnel (Figure 1, #9). Sample loading is achieved using a vacuum-sealed transfer rod (Figure 1, #1), which protects the sample from atmospheric contamination during transfer.

Figure 2 shows the internal configuration of the electrochemical cell. The sample is mounted onto the sample stage holder (Figure 2, #1) and placed on the working electrode (Figure 2, #3), which is located inside the electrolyte reservoir (Figure 2, #5). The reference electrode (Ag/AgCl) (Figure 2, #7) and counter electrode (glassy carbon, GC) (Figure 2, #6) are immersed in the electrolyte, completing the electrochemical circuit. This design enables precise and contamination-free electrochemical treatment of samples prior to surface analysis by ToF-SIMS.

**Figure 2:**
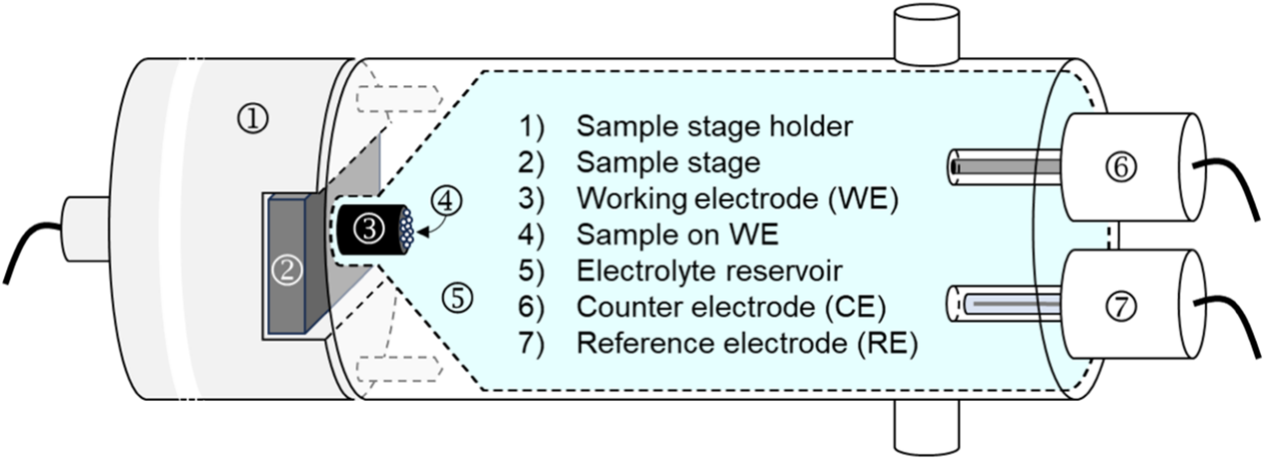
Detailed internal view of the electrochemical cell; the electrolyte reservoir is enclosed by the working and counter/reference electrode sides of the setup.

### Sample Transfer Process

Before transferring the sample, the entire electrochemical cell was purged with argon gas for 30 min to create an oxygen-free environment. Since argon is denser than oxygen, it effectively displaces ambient air. The ToF-SIMS preparation chamber was then brought to atmospheric pressure, and the transfer gate (Figure 1, #10) was opened to move the sample into the EC cell. This pressure adjustment—from 10^–7^ mbar to atmospheric pressure—prevented damage due to differential pressure across the devices. The working electrode was then transferred into the EC cell using the vacuum-sealed transfer rod (Figure 1, #1), ensuring isolation from atmospheric contaminants throughout the process.

### Electrochemical Testing and Postexperiment Handling

1 **Electrochemical Testing:** The catalyst was subjected to OER measurements using a three-electrode configuration (WE, RE, CE) connected to a Metrohm Autolab PGSTAT302N potentiostat as described in the previous publication.^1^
2 **Rinsing and Drying:** After each electrochemical experiment, the WE was rinsed with Milli-Q water and dried by argon gas flow to prevent contamination before further analysis.
3 **Electrolyte Drop-Casting for ToF-SIMS:** To assess possible catalyst leaching, a few drops of used electrolyte were drop-cast onto a clean Si wafer for subsequent ToF-SIMS analysis.
4 **Final Sample Transfer:** The sample was vacuum-transferred back into the ToF-SIMS chamber. The preparation chamber was re-evacuated to 10^– 7^ mbar, and the sample was introduced into the main analysis chamber for after exposure to electrolyte and after electrochemistry characterization.

### ToF-SIMS Surface Analysis

ToF-SIMS measurements were conducted using a ToF IV instrument (IONTOF, Münster, Germany) in static SIMS mode, with a 25 keV Bi^+^ primary ion beam (∼1 pA current). Spectra were acquired in positive ion mode over a 500 × 500 μm^2^ raster area at 128 × 128 pixel resolution. The total primary ion dose density was kept below the static SIMS limit (1 × 10^1–2^ ions/cm^2^). Mass calibration followed the procedure described in Tables S1 and S2, yielding a mass resolving power of ∼6000 at *m*/*z* 29 under static SIMS conditions. Charge compensation was not required due to the conductive glassy carbon substrate and the conductivity of the sample. For statistical reliability, nine different positions were analyzed on the surface of the same sample at three phases: Pristine, after electrolyte exposure, and after cyclic voltammetry.

### Peak Selection Parameters and Preprocessing Conditions

The automated peak search in *SurfaceLab 7.4* (IONTOF GmbH, Münster, Germany) was employed to generate peak lists based on defined criteria. Peaks were selected with a minimum intensity threshold of 100 counts and a minimum signal-to-noise ratio (S/N) of 1.0. A maximum background level of 0.8 was applied. The filter width was adaptively set within a range of 1.0–2.66 ns. Manual selection and wide peak options were not used during this process.

### PCA of ToF-SIMS Spectra

To analyze chemical variations among samples, PCA was applied to the peak lists generated from the ToF-SIMS mass spectra. To ensure consistency across all spectra, a global peak list was created by combining representative peak searches from the pristine, electrolyte-exposed, and postcyclic voltammetry groups (nine positions per group). This global list was then used as the common reference for peak alignment and intensity extraction across all spectra. The raw data, acquired from SurfaceLab as peak list files in ascii format, were processed using a Python script. The preprocessing steps were designed to ensure the comparability and quality of spectral data across all samples prior to analysis.

Prior to PCA, mass calibration was performed on all selected measurement points across the three electrochemical phases to ensure accurate peak alignment. Two sets of mass calibrations were carried out depending on the sample type: one for the nanoparticle’s surfaces (Table S1), and the other for electrolyte analysis after electrochemical treatment (Table S2).

Following calibration, the data were preprocessed at the variable (*m*/*z*) level. The variables were scaled by dividing by their standard deviation to ensure equal contribution to the PCA and mean-centered. PCA loadings were taken directly from the scaled and centered data without reverse scaling.

## Results and Discussion

The ToF-SIMS mass spectrum of the pristine catalyst surface (Figure 3) provides a chemical fingerprint of the catalyst surface, highlighting prominent ions originating from Nafion, PVP, KOH electrolyte, and the Fe–Ni oxide nanoparticle. The molecular origins of the annotated fragments in Figure 3 are as follows: CF^+^, C_2_F_4_^+^, and C_3_F_5_^+^ originate from Nafion; CHO^+^, C_2_H_4_^+^, C_3_H_5_^+^, C_3_H_7_^+^ and C_5_H_9_^+^ are attributed to PVP; K^+^ is derived from the KOH electrolyte; and FeH^+^ and NiH^+^ originate from the FeNi catalyst. These fragments, color-coded in Figure 3 for visual clarity, represent the pristine catalysts surface.

**Figure 3:**
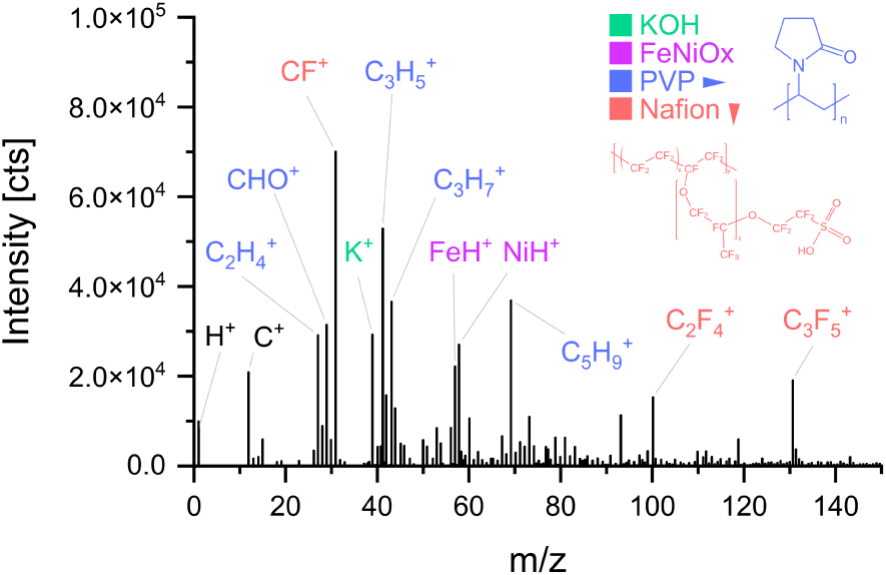
ToF-SIMS spectrum of Fe:Ni nanoparticles (2:3 atomic ratio). Color-coded peak assignments indicate fragments from PVP (blue), Nafion (orange), FeNiO_*x*_ nanoparticles (purple), and KOH electrolyte (green), providing a fingerprint of surface composition.

To evaluate and resolve surface changes over the three experimental stages, PCA was applied to ToF-SIMS spectra across the three experimental phases: pristine, after electrolyte exposure, and after cyclic voltammetry. The PCA scores plot (Figure 4) revealed a clear separation of the three data groups, indicating distinct surface compositions at each phase.

**Figure 4:**
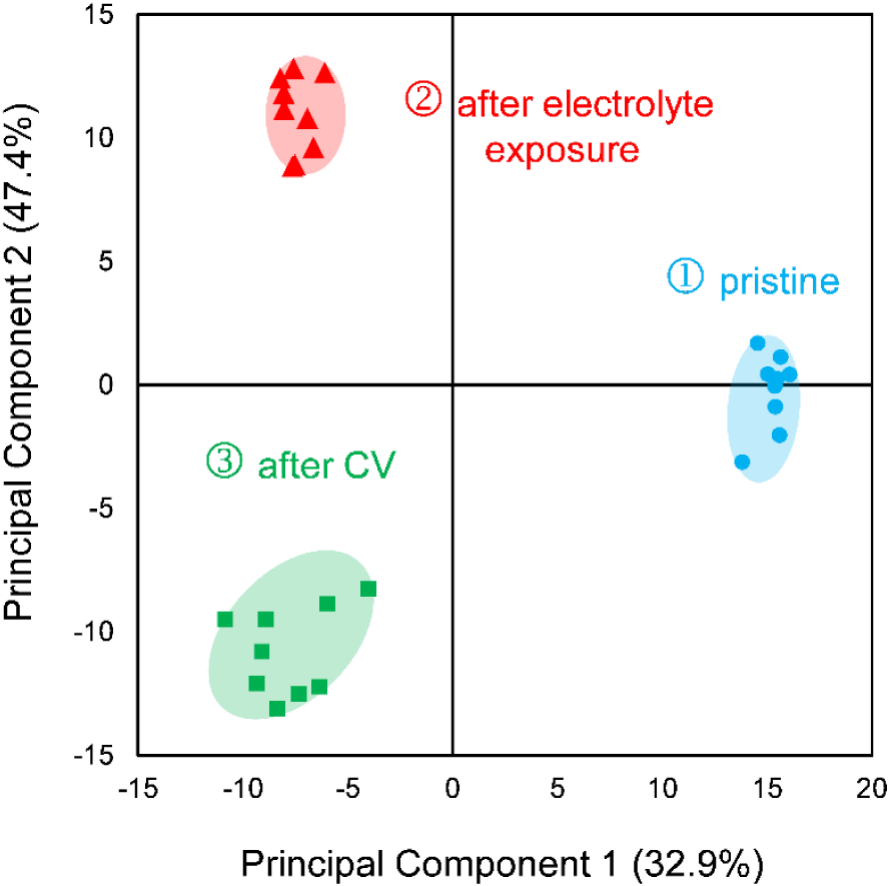
PCA scores plot of Fe:Ni nanoparticles (2:3 atomic ratio), showing clear group separation across experimental conditions: pristine (blue), after electrolyte exposure (red), and after cyclic voltammetry (CV) (green). Each group contains 9 measurements.

Principal Component 1 (PC1, 47.4% of the variance) separates the pristine samples from those exposed to KOH, while PC2 (32.9% of the variance) further distinguishes the samples after exposure to electrolyte from after electrocatalysis, capturing additional chemical changes induced by electrochemical activation.

Altogether, PC1 and PC2 explain 80.3% of the total variance in the data set, indicating progressive differentiation among sample surfaces, likely due to chemical changes during electrochemical treatment, as reflected by the PCA score separation. These results support the hypothesis that the surface chemistry evolves first due to alkaline electrolyte exposure, and then more significantly through electrocatalytic reaction.

Positive PC1 loadings (Figure 5) are associated with the pristine state and include fragments such as C_2_H_5_O^+^, C_6_H_11_^+^, C_2_H_6_N^+^, C_3_H_8_O_2_^+^, C_3_H_8_N_2_^+^, C_3_H_7_N_2_^+^, C_3_H_5_O^+^, C_9_H_17_N_2_^+^, C_10_H_19_O^+^, C_10_H_20_O^+^, C_4_H_10_NO^+^, C_2_H_8_N^+^, C_10_H_20_N^+^, and C_3_H_8_NO^+^. The ions identified as significant in the PCA loadings were validated by ranking the loadings and cross-checking the corresponding peaks in the global peak list (SurfaceLab), which confirmed the same differences between the sample groups as seen in the PCA scores. These fragments are consistent with organic and nitrogen-containing species, particularly PVP ligands introduced during nanoparticle synthesis. Their strong contribution to early stage samples suggests that the catalyst surface remains ligand-covered prior to electrochemical activation.

**Figure 5:**
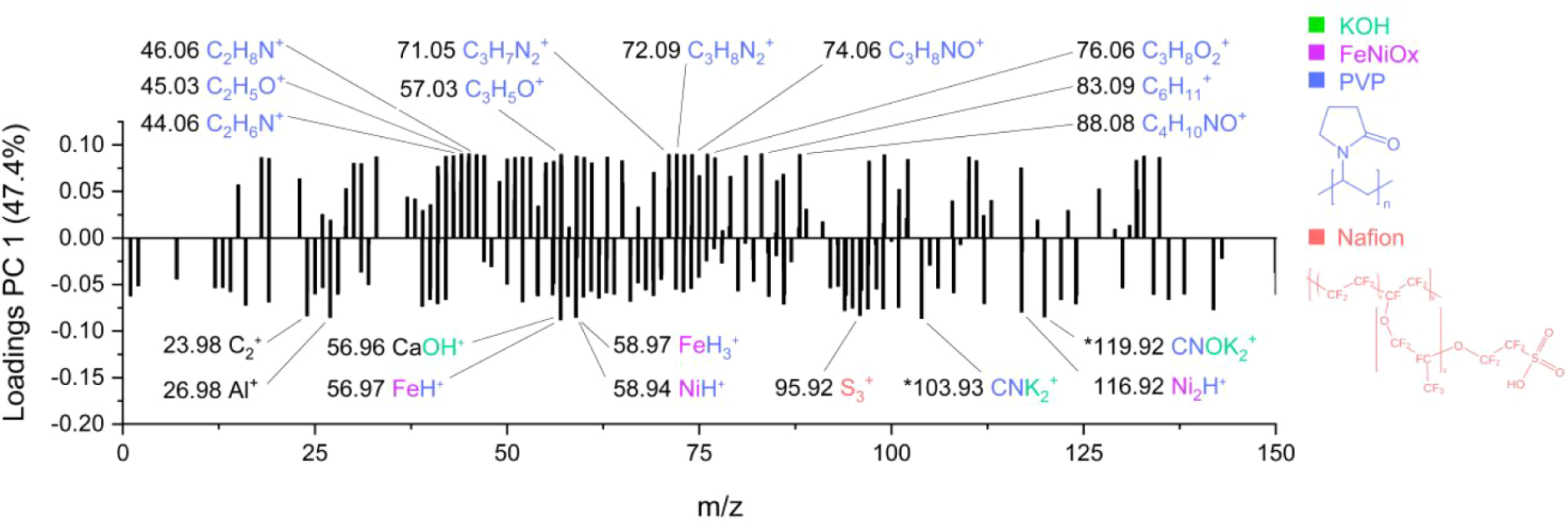
PCA loadings plot of the particle surface analysis corresponding to PC1 for Fe:Ni nanoparticles (2:3 ratio). Positive loadings are associated with pristine samples, while negative loadings correspond to samples before and after electrochemical treatment. Labeled peaks indicate fragments from PVP, Nafion, FeNiO_*x*_, and electrolyte-related species. Peaks marked with an asterisk (*) have multiple possible assignments.

In contrast, negative PC1 loadings (Figure 5) include fragments assigned to electrolyte and Nafion components and metal-related species. These ions appear after KOH exposure and persist through electrochemical cycling, reflecting the chemical and electrochemical transformations occurring at the catalyst surface. For readability, only the most significant peaks are annotated in the PCA loading plots. The additional peaks correspond mainly to isotopic variants, adducts (e.g., +H^+^, +K^+^), or homologous fragments within the same chemical families. These peaks contribute coherently with the annotated ions to the observed separation in the PCA scores.

For a complete list of assigned fragments and their intensities, please refer to Tables S3 and S4 in the SI. Note that some fragments have multiple possible assignments, and the most probable ones are indicated by the lowest deviation values.

The loadings plot for PC2 (Figure 6) helps interpret the chemical drivers behind the observed sample separation before and after catalysis. Positive PC2 loadings are dominated by fragments such as C_2_H_3_N^+^, *C_6_H_6_^+^, C_3_H_6_N^+^, C_3_H_3_O^+^, CH_2_N^+^, CH_2_NO^+^, C_2_H_2_^+^, *C_7_H_7_^+^, C_5_H_6_^+^, C_5_H_7_^+^, C_4_H_8_N^+^, CO^+^, C_5_H_8_^+^, C_4_H_6_^+^, and C_3_H_6_N^+^ which are attributed to PVP ligand fragments. Their strong presence at positive PC2 loadings reflects the ligand-covered state of the catalyst surface before electrochemical activation. Electrochemical cycling promotes their displacement via ligand exchange and oxidation processes, leading to the exposure of electrochemically active regions.

**Figure 6:**
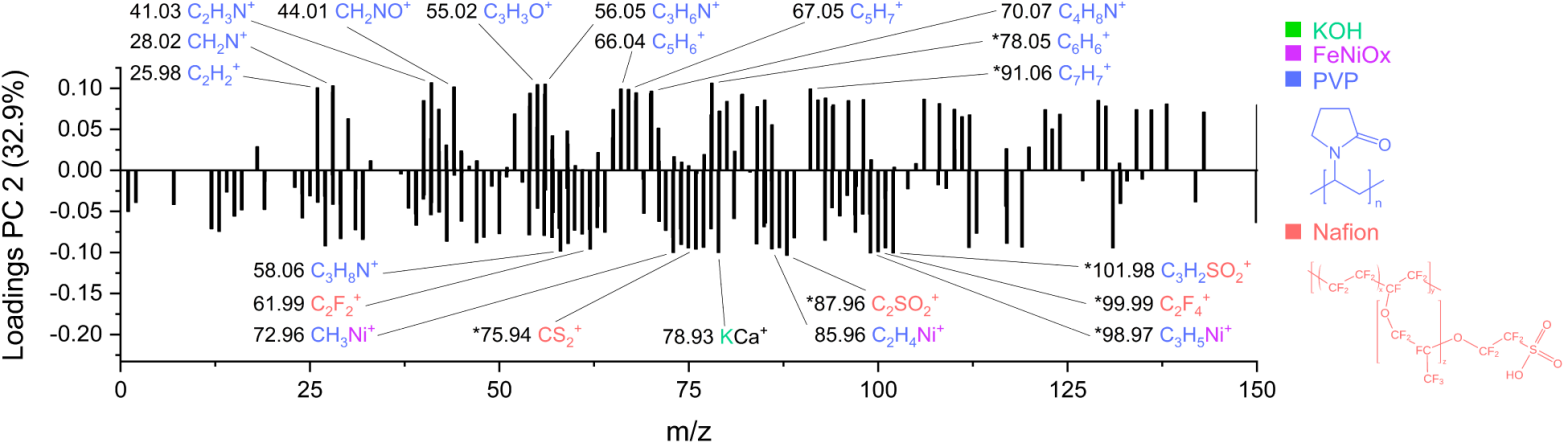
PCA loadings plot corresponding to PC2 for Fe:Ni nanoparticles (2:3 ratio). Positive loadings are associated with pristine samples, while negative loadings correspond to samples before and after electrochemical treatment. Labeled peaks indicate fragments from PVP, Nafion, FeNiO_*x*_, and electrolyte-related species. Peaks marked with an asterisk (*) have multiple possible assignments.

Two of the fragments marked with asterisks (C_6_H_6_^+^ and C_7_H_7_^+^) have multiple possible structural assignments. These ions are discussed in detail in the SI (Table S5), which includes the full list of all fragments, along with their *m*/*z* values, signal intensities, deviations, and alternative structural candidates to support accurate peak interpretation.

Conversely, negative PC2 loadings, which are characteristic of the after-electrocatalysis phase, are dominated by fragments such as *C_2_SO_2_^+^, *C_3_H_5_Ni^+^, *C_3_H_2_SO_2_^+^, CH_3_Ni^+^, *C_2_F_4_^+^, C_3_H_8_N^+^, C_2_F_2_^+^, *CS_2_^+^, C_2_H_4_Ni^+^, *FeF^+^, C_3_HSO_2_^+^, C_3_F_5_^+^, C_2_HSNO^+^, and C_3_F_4_^+^ which are attributed to FeNiO_*x*_ species and binder-derived fragments Nafion, as well as ink solvents such as ethanol and water.

The ions marked with asterisks (C_2_SO_2_^+^, C_3_H_5_Ni^+^, C_3_H_2_SO_2_^+^, C_2_F_4_^+^, FeF^+^) have ambiguous structural assignments. These are discussed in detail in the SI (Table S6).

This shift in surface composition indicates enhanced exposure of the FeNi active phase and the presence of Nafion-derived species at the catalyst–electrolyte interface after OER cycling. The detection of multiple sulfonate- and fluorocarbon-based fragments suggests molecular changes in the binder environment, likely influenced by prolonged exposure to alkaline conditions and applied potential.

These findings support the conclusion that the PVP ligands are removed from the nanoparticle surface during electrocatalysis, thereby exposing the underlying FeNiO_*x*_ and Nafion, both of which contribute to enhanced OER activity.

### Electrolyte Analysis: Insights from ToF-SIMS and PCA

To validate the conclusion from the catalyst surface analysis that components of the catalyst system leach into the electrolyte during electrochemical processing, ToF-SIMS was performed on drop-casted electrolyte samples collected after each treatment phase. Among the electrolyte samples that were in contact with the catalyst (after electrolyte exposure, and after cyclic voltammetry), characteristic signals related to components of the catalyst system—PVP, Nafion, and FeNiO_*x*_—were detected. Observed fragments included C_2_H_5_^+^, C_2_H_2_O^+^, C_2_H_5_O^+^, C_2_F^+^, NiO^+^, NiOH^+^, CH_3_SONi^+^, FeSNH_3_^+^, and Ni_2_OH^+^, providing direct evidence of catalyst-derived species leaching into the electrolyte. These fragments were absent in the group containing only KOH, confirming their origin from the catalyst system.

To focus specifically on the impact of electrocatalysis, PCA was performed on the electrolyte ToF-SIMS spectra excluding the pristine electrolyte, thereby comparing the electrolyte before and after electrochemical cycling. The scores plot (Figure 7) shows a clear separation along Principal Component 1 (PC1, 55.4% explained variance) between the two experimental groups. This indicates a change in the chemical composition of the electrolyte induced by the electrocatalytic reaction.

**Figure 7:**
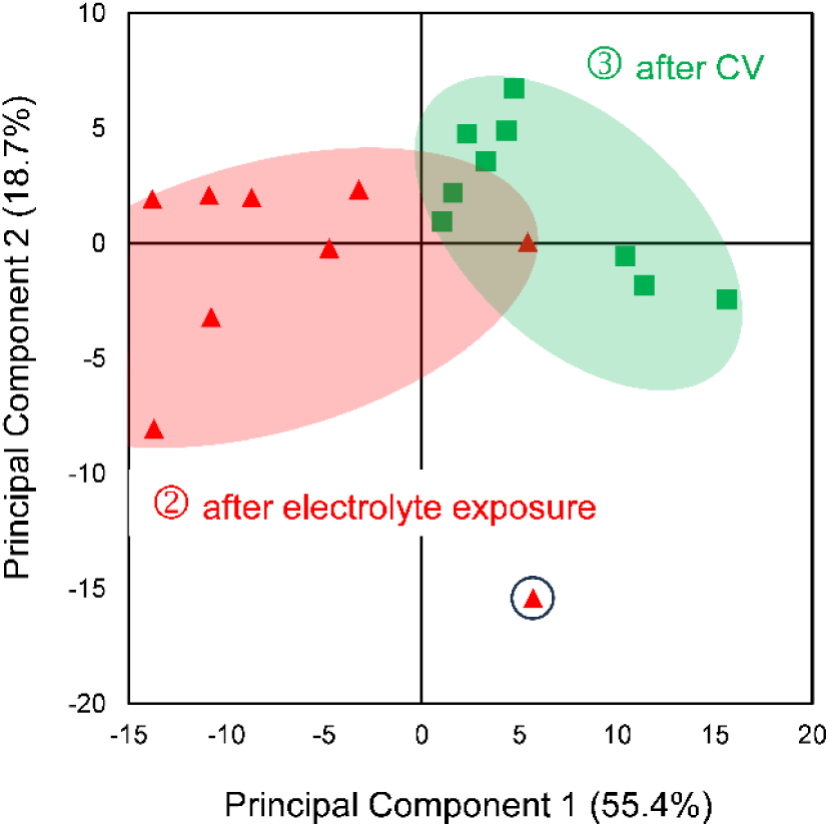
PCA scores plot of electrolyte compositions before and after electrochemical treatment, showing distinct group separation: after electrolyte exposure (red) and after cyclic voltammetry (CV) (green). Each group contains 9 measurements.

Notably, PC2 (18.7% explained variance) carried no relevant information, suggesting that PC1 accounts for the principal distinction between groups.

The PC1 loadings plot (Figure 8) highlights the chemical fragments responsible for the group separation. Most of the positive loadings, corresponding to the postcatalysis electrolyte are associated with PVP-related fragments, indicating an increased amount of PVP in the electrolyte after the CV cycles. This finding aligns with the hypothesis that the polymer ligand on the surface of the catalyst is detached and released into the electrolyte during electrochemical operation. In contrast, fragments such as K_2_OH^+^, ^41^KKOH^+^, K_2_C_2_H^+^, CH_3_SOFe^+^, S_2_NO_3_H^+^, and Ni_2_OH^+^ with strong negative PC1 loadings are more prominent in the electrolyte before electrocatalysis. These ions likely reflect early stage leaching of a metal–organic species from the catalyst composite during immersion.

**Figure 8:**
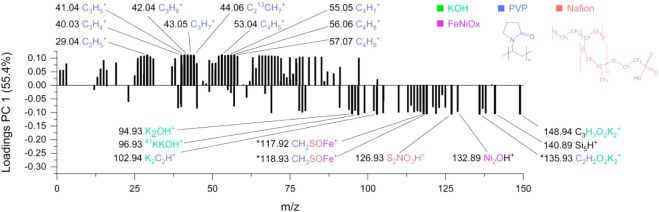
Loadings plot of PC1 from the electrolyte analysis. Positive loadings correlate to the postcatalysis stage, indicating the release of PVP into the electrolyte after or during electrocatalysis. Negative loadings correlate to the precatalysis stage, fragments from all catalyst components could be found. Peaks marked with an asterisk (*) have multiple possible assignments.

The detailed assignments for the PC1-related signals are presented in two supporting tables provided in the Supporting Information: one for positive-loading PC1 fragments and one for negative-loading PC1 fragments. These tables (Tables S7 and S8) include each ion’s *m*/*z*, intensity, deviation, and explained variance, and are sorted by deviation to support confident interpretation. In both tables, fragments with more than one possible molecular assignment are marked with an asterisk (*). These ambiguous cases are discussed further in the SI, where plausible structures are proposed based on chemical context and peak deviation.

The results emphasize that not only does the surface chemistry of the catalyst evolve during catalysis, but species (including ligands and metal complexes) are released into the electrolyte.

## Conclusions

This study provides a comprehensive investigation of the surface evolution of Fe–Ni oxide nanocatalysts under OER conditions, using a unique integrated electrochemical-ToF-SIMS setup combined with PCA analysis. Through multistage surface analysis, we demonstrate that the electrocatalytic process induces distinct chemical changes at the nanoparticle–electrolyte interface. PCA was applied here as a qualitative tool to reveal discriminating ion signals. Due to matrix effects and ionization efficiencies in ToF-SIMS, peak intensities from PCA loadings cannot be used directly for quantification.

Before electrochemical activation, the catalyst surface is dominated by organic and nitrogen-containing fragments (PVP-derived ligands), as shown by positive PC1 and PC2 loadings. These surface ligands that are introduced during nanoparticle synthesis, were found to persist on the surface, forming a dense organic layer that potentially hinders active site exposure. However, after the first cycles measurements revealed their displacement, with a corresponding rise in ligand fragments in the electrolyte phase, confirming electrochemical removal and leaching.

PCA scores and loadings plots validate these chemical shifts, with PC1 and PC2 capturing the transition from a ligand-saturated to a ligand-depleted and metal-restructured surface.

The schematic illustration (Figure 9) supports the mechanistic interpretation derived from surface and electrolyte analyses. Prior to electrochemical activation, FeNiO_*x*_ nanoparticles are embedded within a composite interphase consisting of residual PVP ligands and Nafion domains, forming a stratified, relatively uniform interface with the alkaline electrolyte. After catalysis, the interface becomes more chemically heterogeneous, with fewer surface-bound ligands and an altered Nafion arrangement, highlighting the activation-driven exposure of electrochemically active sites.

**Figure 9:**
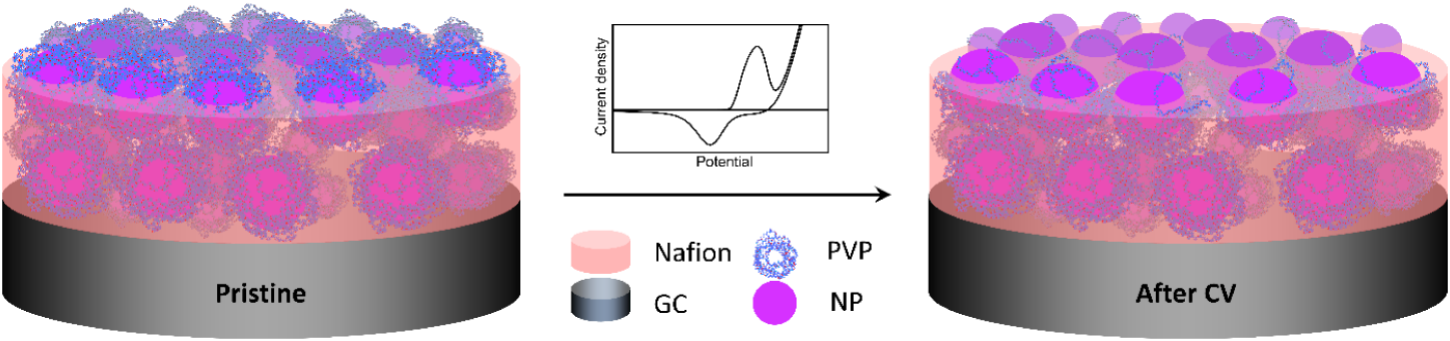
Schematic of the FeNiO_*x*_–electrolyte interface before and after CV measurement. During OER, a portion of the PVP ligands is removed, leading to partial exposure of the nanoparticle catalyst surface.

Together, these findings underscore the critical role of ligand dynamics and interfacial evolution in optimizing catalyst function. By correlating spectral changes with electrochemical treatment, we established a powerful strategy to monitor surface activation mechanisms offering new insights into catalyst conditioning. As an outlook, the workflow applied here could also be extended to study condition-dependent surface changes. Variations in CV parameters (e.g., cycle number, potential range) or electrolyte treatments (e.g., KOH concentration, exposure time) may likewise lead to detectable spectral differences, particularly when such changes influence the distribution of surface fragments.

## Supplementary Material



## Notes

Two figures with the sample holder and the electrochemical cell stage used in this study, together with two tables showing calibration data and five tables showing the lists of specific key fragment ions relevant to this paper (PDF)
