# Supplementary material for: Probing Surface Changes in Fe–Ni Oxide Nanocatalysts with a ToF-SIMS-Coupled Electrochemistry Setup and Principal Component Analysis
Source: Anal Chem. 2025 Dec 16;97(51):28173–80. doi: 10.1021/acs.analchem.5c03894 (PMC12756852; doi:10.1021/acs.analchem.5c03894)
Supplement: Supplementary file 1 [file ac5c03894_si_001.pdf]

Supporting Information

Probing Surface Changes in Fe-Ni Oxide Nanocatalysts with a ToF-SIMS-coupled Electrochemistry Setup and Principal Component Analysis

Heydar Habibimarkani[a], Jörg Radnik[a,*], Vasile-Dan Hodoroaba[a,*], Elisabeth John[a]

[a] Federal Institute for Materials Research and Testing (BAM), Unter den Eichen 87, 12205 Berlin, Germany

*Corresponding authors: Dan.Hodoroaba@bam.de; Joerg.Radnik@bam.de

Table of contents:

- Figure S1 and Figure S2……………………………………………...…S2
- Tables S1 to Table S8…………………………………………….S3 to S7

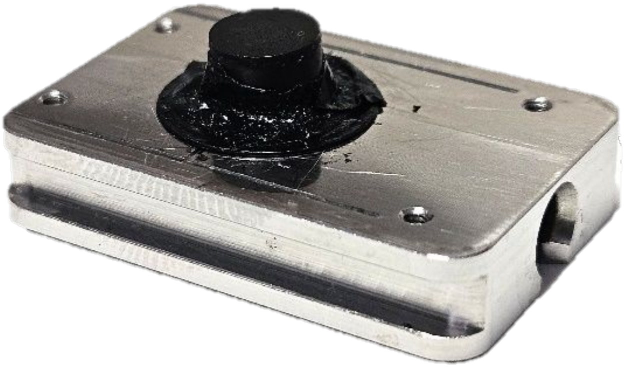

Figure S1. *The sample holder*

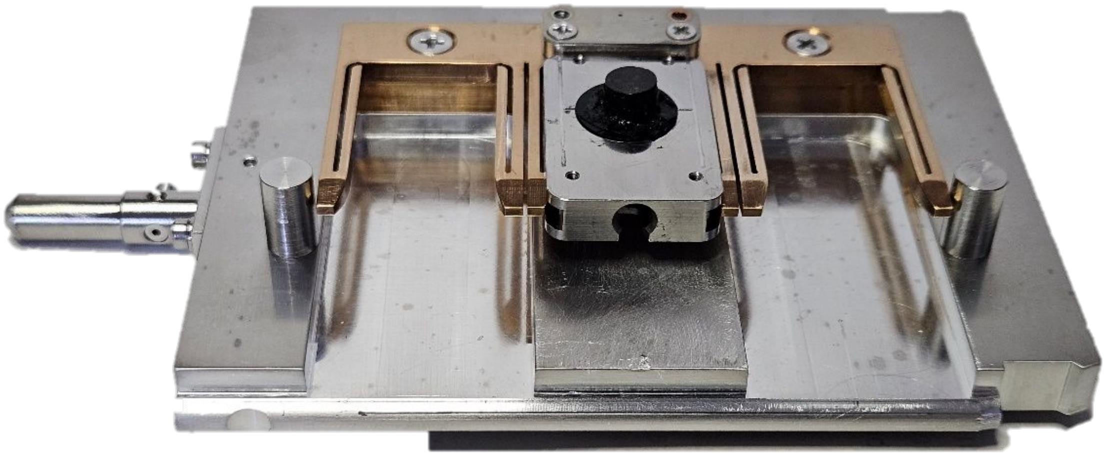

Figure S2. *Electrochemical cell stage*

Table S1. *Calibration ions for nanoparticle surface analysis*

Ion	Mass (m/z)	Mass Deviation (ppm)
CH_2^+	14.015101	-12.1
CH_3^+	15.022927	7.3
$C_4H_9^+$	57.069877	-1.9
CF^+	68.994661	19.4
$C_3F_2^+$	73.996258	1.9
$C_3F_3^+$	92.994661	-9.0
$C_2N_2O_3^+$	99.990343	7.2
$C_2F_5^+$	118.991467	-10.0
$C_3F_5^+$	130.991467	-17.9
$C_5F_5^+$	154.991467	6.0
$C_8OF_3^+$	168.989576	7.7
$C_4F_7^+$	180.989576	0.6

Table S2. *Calibration ions for electrolyte analysis after electrochemistry*

Ion	Mass (m/z)	Mass Deviation (ppm)
CH_3^+	15.022927	-9.3
$C_2H_5^+$	29.038577	28.7
$C_3H_7^+$	43.054227	-34.2
$C_4H_7^+$	55.054227	-15.4
CH_2OK^+	68.973723	-1.7
K_2O^+	93.921780	-5.5
$NiO4^+$	121.914458	12.3
$C_2H_2O_2K_2^+$	135.932345	11.7
CO_3K3^+	176.875316	37.3
$K_3SO_4^+$	212.842301	8.3
$Si_3H_5O_9^+$	232.923588	-39.5

Table S3. *List of key fragment ions with high positive PC1 loadings, primarily associated with pristine Fe:Ni (2:3) nanoparticles. The table includes m/z values, signal intensities, proposed chemical assignments, deviation from theoretical masses, and percentage of explained isotopic pattern.*

No.	m/z	Area (cts)	Assignment(s)	Deviation (ppm)	Explained (%)
1	45.0342	985	$C_2H_5O^+$	-9.6	100
2	83.071	1472	$C_6H_{11}^+$	25.57	100
3	44.056	2673	$C_2H_6N^+$	-2.03	100
4	76.0547	54	$C_3H_8O_2^+$	21.72	100
5	72.0859	313	$C_3H_8N_2^+$	48.38	100
6	71.0541	781	$C_3H_7N_2^+$	-11	100
7	57.0344	1184	$C_3H_5O^+$	7.18	100
8	153.1223	38	$C_9H_{17}N_2^+$	-4.52	100
9	155.1559	58	$C_{10}H_{19}O^+$	40.06	100
10	156.1489	38	$C_{10}H_{20}O^+$	-6.9	100
11	88.0758	17	$C_4H_{10}NO^+$	-19.1	100
12	46.0642	32	$C_2H_8N^+$	15.8	100
13	154.1454	690	$C_{10}H_{20}N^+$	-88.07	100
14	74.0574	1143	$C_3H_8NO^+$	23.16	100
15	44.0579	9489	$C_2H_6N^+$	-2.0	100

Table S4. *List of key fragment ions with low positive PC1 loadings, primarily associated with before and after electrochemical treatment. The table includes m/z values, signal intensities, proposed chemical assignments, deviation from theoretical masses, and percentage of explained isotopic pattern.*

No.	m/z	Area (cts)	Assignment(s)	Deviation (ppm)	Explained (%)
1	56.9721	128	FeH^+	-51.27	100
2	103.9293	636	CNK_2^+	-5.7	100
			$FeSNH_2^+$	40.2	100
3	26.9815	4048	Al^+	20.7	100
4	58.9675	584	FeH_3^+	-29.32	100
5	119.9233	273	$CNOK_2^+$	-12.97	100
			$FeSNOH_2^+$	26.37	100
			KSO_3H^+	-37.8	100
			$CH_2SO_2Ni^+$	48.62	100
6	58.9412	1477	NiH^+	-45.8	100
7	23.9847	80	C_2^+	9.23	100
8	56.9636	2061	$CaOH^+$	-33.7	100
9	95.9209	143	S_3^+	54.55	100
10	157.8822	4708	$K_2SO_3^+$	-9.44	100
11	116.9243	91	Ni_2H^+	-69.18	97.7
12	93.9222	922	K_2O^+	2.93	100
13	141.8863	298	$K_2SO_2^{2+}$	-17.4	100
14	176.8837	481	$CO_3K_3^+$	47.2	100
15	96.9276	47833	K_2F^+	17.9	100
16	98.9247	7222	$K^{41}KF^+$	13.3	95.9
17	94.9271	436	K_2OH^+	-26	100
18	100.9193	264	CH_3SFe^+	45.2	100
19	174.8859	195	$K_2SO_4H^+$	-2.7	100
20	38.9644	224546	K^+	32	100

Table S5. *List of key fragment ions with high positive PC2 loadings in electrolyte, primarily associated with before electrochemical treatment Fe:Ni (2:3) nanoparticles. The table includes m/z values, signal intensities, proposed chemical assignments, deviation from theoretical masses, and percentage of explained isotopic pattern.*

No.	m/z	Area (cts)	Assignment(s)	Deviation (ppm)	Explained (%)
1	41.0254	3469	$C_2H_3N^+$	-16.9	100
2	78.0452	1057	$C_6H_6^+$	-14.96	100
			$C_3H_7OF^+$	-29.6	100
3	56.0538	10505	$C_3H_6N^+$	-5.7	100
4	55.0173	18.96	$C_3H_3O^+$	-7	100
5	28.0195	2654	CH_2N^+	45.7	100
6	44.0129	431	CH_2NO^+	-7.4	100
7	25.976	2809	$C_2H_2^+$	1.38	100
8	91.0548	1470	$C_7H_7^+$	-8.9	100
			$C_2H_7N_2O_2^+$	35.32	100
			$C_4H_8OF^+$	-21.42	100
			$C_4H_{11}S^+$	-45.88	100
9	66.0442	816	$C_5H_6^+$	-13.76	100
10	67.0521	4716	$C_5H_7^+$	-22.90	100
11	70.0716	1340	$C_4H_8N^+$	23	100
12	27.994	157	CO^+	15	100
13	68.0586	932	$C_5H_8^+$	-23	100
14	54.0414	4551	$C_4H_6^+$	-67.4	100
15	56.0118	5449	$C_3H_6N^+$	-5.7	100

Table S6. *List of key fragment ions with low positive PC2 loadings in electrolyte, primarily associated with after electrochemical treatment Fe:Ni (2:3) nanoparticles. The table includes m/z values, signal intensities, proposed chemical assignments, deviation from theoretical masses, and percentage of explained isotopic pattern.*

No.	m/z	Area (cts)	Assignment(s)	Deviation (ppm)	Explained (%)
1	87.9596	724	$C_2SO_2^+$	-19.21	100
			$KSNH_3^+$	-24.06	100
2	98.9736	4545	$C_3H_5Ni^+$	-8.23	100
			$H_3SO_4^+$	-15.62	100
			C_3HSNO^+	-42.7	100
3	101.9782	1677	$C_3H_2SO_2^+$	1.68	100
			CSN_3O^+	14.84	100
4	72.9556	1235	CH_3Ni^+	-40.64	100
5	78.9268	292	KCa^+	-4.98	100
6	99.9926	42271	$C_2F_4^+$	-3.51	100
			$C_4HO_2F^+$	-27.91	100
			$C_2N_2O_3^+$	23.75	100
7	58.0647	8645	$C_3H_8N^+$	-12.35	100
8	61.9943	2262	$C_2F_2^+$	-31.66	100
9	75.9443	807	CS_2^+	6.07	100
			NiH_2O^+	-17.26	100
10	85.9649	1891	$C_2H_4Ni^+$	-13.51	100
11	74.9345	1699	FeF^+	22.98	100
			$NiOH^+$	-40.3	100
12	100.9686	1092	$C_3HSO_2^+$	-5.9	100
13	130.9906	38349	$C_3F_5^+$	-6.9	100
14	86.9752	274	C_2HSNO^+	-27.3	100
15	111.992	4445	$C_3F_4^+$	-9.9	100
			$C_3N_2O_3^+$	14.9	100

Table S7. *List of key fragment ions with high positive PC1 loadings in electrolyte, primarily associated with after electrochemical treatment Fe:Ni (2:3) nanoparticles. The table includes m/z values, signal intensities, proposed chemical assignments, deviation from theoretical masses, and percentage of explained isotopic pattern.*

No.	m/z	Area (cts)	Assignment(s)	Deviation (ppm)	Explained (%)
1	53.0373	10306	$C_4H_5^+$	-26.0	100
2	41.0368	94661	$C_3H_5^+$	-39.5	100
3	29.0394	87993	$C_2H_5^+$	32.3	100
4	42.0433	14346	$C_3H_6^+$	-65.9	100
5	57.0701	24217	$C_4H_9^+$	4.2	100
6	43.0528	65162	$C_3H_7^+$	-29.35	100
7	55.0534	28596	$C_4H_7^+$	-15.50	100
8	56.0608	6439	$C_4H_8^+$	-22.65	100
9	40.027	7990	$C_3H_4^+$	-80.61	100
10	44.0558	3048	$C_2{}^{13}CH_7^+$	-38.98	100
11	29.0019	118243	CHO+	-9.83	100
12	54.0448	5925	$C_4H_6^+$	-30.72	100
13	67.0538	2462	$C_5H_5^+$	-11.28	100
14	66.0456	984	$C_5H_6^+$	-18.82	100
15	68.061	1248	$C_5H_8^+$	-18. 2	100

Table S8. *List of key fragment ions with low positive PC1 loadings in electrolyte, primarily associated with before electrochemical treatment Fe:Ni (2:3) nanoparticles. The table includes m/z values, signal intensities, proposed chemical assignments, deviation from theoretical masses, and percentage of explained isotopic pattern.*

No.	m/z	Area (cts)	Assignment(s)	Deviation (ppm)	Explained (%)
1	96.9287	9688	$K^{41}KOH^+$	6.0	73.5
2	132.8853	623	Ni_2OH^+	84	37.4
3	102.9352	6475	$K_2C_2H^+$	1,59	100
4	135.9339	2924	$C_2H_2O_2K_2^+$	3.1	100
			$C_2S_2O_3^+$	32.63	100
			$CS_2N_2O_2^+$	-50.00	100
5	126.9343	1062	$S_2NO_3H^+$	-46.97	100
6	148.9442	2331	$C_3H_3O_2K_2^+$	21.7	100
7	94.9315	48127	K_2OH^+	16.5	100
8	117.9199	227	CH_2SOFe^+	20.2	100
			CH_2SNNi^+	-51,2	100
9	118.9289	889	CH_3SOFe^+	31.1	100
			CH_3SNNi^+	-40.91	100
10	140.892	1093	Si_5H^+	-5.3	100
11	121.9158	5599	$K_2O_2C^+$	-8.8	100
12	137.9114	301	KSO_3F^+	-55.6	100
13	104.9326	1083	FeO_3H^+	57.1	100
14	68.9736	366	CH_2OK^+	-0.2	100
15	98.9267	634	$K^{41}KF^+$	30.9	100
